# Identification of tumor tissue in thin pathological samples via femtosecond laser-induced breakdown spectroscopy and machine learning

**DOI:** 10.1038/s41598-023-36155-8

**Published:** 2023-06-08

**Authors:** Cristian Sarpe, Elena Ramela Ciobotea, Christoph Burghard Morscher, Bastian Zielinski, Hendrike Braun, Arne Senftleben, Josef Rüschoff, Thomas Baumert

**Affiliations:** 1 Institut für Physik, Universität Kassel, Heinrich-Plett-Str. 40, 34132 Kassel, Germany; 2 Institut für Pathologie Nordhessen, Germaniastr. 7, 34119 Kassel, Germany

**Keywords:** Cancer, Breast cancer, Optical spectroscopy, Ultrafast lasers

## Abstract

In the treatment of most newly discovered solid cancerous tumors, surgery remains the first treatment option. An important factor in the success of these operations is the precise identification of oncological safety margins to ensure the complete removal of the tumor without affecting much of the neighboring healthy tissue. Here we report on the possibility of applying femtosecond Laser-Induced Breakdown Spectroscopy (LIBS) combined with Machine Learning algorithms as an alternative discrimination technique to differentiate cancerous tissue. The emission spectra following the ablation on thin fixed liver and breast postoperative samples were recorded with high spatial resolution; adjacent stained sections served as a reference for tissue identification by classical pathological analysis. In a proof of principle test performed on liver tissue, Artificial Neural Networks and Random Forest algorithms were able to differentiate both healthy and tumor tissue with a very high Classification Accuracy of around 0.95. The ability to identify unknown tissue was performed on breast samples from different patients, also providing a high level of discrimination. Our results show that LIBS with femtosecond lasers is a technique with potential to be used in clinical applications for rapid identification of tissue type in the intraoperative surgical field**.**

## Introduction

Surgery remains the main line of attack to eradicate cancer discovered in its early stages. Most of the newly diagnosed solid tumors are removed by surgery, hoping for a complete cure or at least prolonging the patient life expectancy^1^. Cancer cells left after the operation (e.g., by positive margins of the resection specimen) can generate local recurrences or metastases over time, being one of the key factors determining a patient’s survival rate. In many cases, subsequent surgical interventions are necessary to remove the newly formed neoplastic tissue, or adjuvant therapies (radiotherapy or chemotherapy) are needed, which have many side effects. The surgery results are predominantly determined by the medical team’s experience performing the oncological intervention: the aim is to completely remove the malignant cells (to prevent further recurrences) and preserve as much tissue as possible from the affected organ, not to degrade its functionality. In practice, the oncological safety margins vary between 2 mm and 1 cm, depending on the type of cancer and the location of the tumor^2^. High-precision localization of the tumor is of crucial importance in the success of the operation. The surgical team can use the information obtained before the operation from imaging techniques (magnetic resonance tomography, x-ray computed tomography or ultrasound imaging), but in the operative field, the decisions are mainly based on visual and tactile information. Many times, to decide if the malignant tissue has been completely removed, the intraoperative pathological examination on a frozen specimen is used. This procedure requires several tens of minutes and, in the case of uncertainty, would significantly increase the operation time, increasing the risk of complications. For this reason, an alternative or complementary technique with a quick and precise establishment of the type of tissue operated on is highly desirable.

In recent years, several innovative techniques have been investigated for in-vivo analysis. Mass spectroscopy techniques, in which mass/charge values are measured for different molecular fragments resulting from the local decomposition of the tissue, have already been tested in-vivo to identify different types of cancer^3–5^. In parallel with these, optical techniques, such as optical coherence tomography^6,7^, Raman spectroscopy^8–10^ and Laser-Induced Breakdown Spectroscopy (LIBS), have been investigated due to their portability and high spatial precision. Even if the first attempts to use LIBS to detect cancerous tissue date back nearly two decades^11^, the development in recent years of Machine Learning (ML) algorithms for interpreting a large volume of experimental data has intensified these studies^12^. The LIBS technique analyzes the emission spectra of the plasma created by lasers focused on the surface of materials. It has the advantage of producing quick results on a wide variety of samples that do not require complicated pre-treatment. In the LIBS process, the material is ionized and plasma is produced, which upon cooling will emit radiation specific to the chemical elements existing in the material. Many studies that try to identify different types of malignant tissue are carried out using nanosecond lasers^12–22^, producing high temperature plasma with significant thermal damage to the sample and a decrease in spatial resolution^23^. In previous studies, we have shown that femtosecond (fs) pulses can be used for precision in situ/in vivo LIBS analysis of biological tissue^23^ and technical samples^24^, allowing a spatial resolution on the order of microns and below ^25^. Applications of fs-LIBS on different biological tissues are presented in several studies (Ref.^26^ and references therein), but its use in the detection of cancerous tissue has been less investigated^12,27–29^.

This article presents our results in identifying liver and breast cancer in standard human pathological samples using fs-LIBS and ML algorithms. Using the advantages of ultrashort laser pulses, we could record atomic and molecular emission spectra following ablation on very thin samples with thicknesses of a few microns. The type of tissue from which the recorded spectrum originates (used in training and evaluation of ML algorithms) could be identified by direct comparison with the results of the pathological analysis on adjacent cuts. To our knowledge, this type of analysis on samples of micrometric thickness was performed only in one recent study on gastrointestinal tumor tissue by using ns-laser pulses^22^, but the spectra presented in this publication have a strong spectral contribution from the substrate, with many emission lines overlapping with the ones coming from the biological tissue. Using ultrashort laser pulses and a high-purity quartz substrate, we performed ablation with a high spatial resolution and low collateral damage, and the recorded spectra contain only contributions from the sample.

The paper is structured as follows: the next section presents the experimental procedure and sample preparation information. Then, fs-LIBS and ML are tested with liver tissue to observe if the spectra obtained from very thin pathological samples can be used to identify the tissue type. Also, in this section, the reproducibility of the measurements is investigated through comparisons between the data recorded on different days. The last section presents the results obtained from breast cancer and how the algorithms already trained on some patients can be used to detect cancer cells in a different patient.

## Methods

### Experimental setup

**Figure 1:**
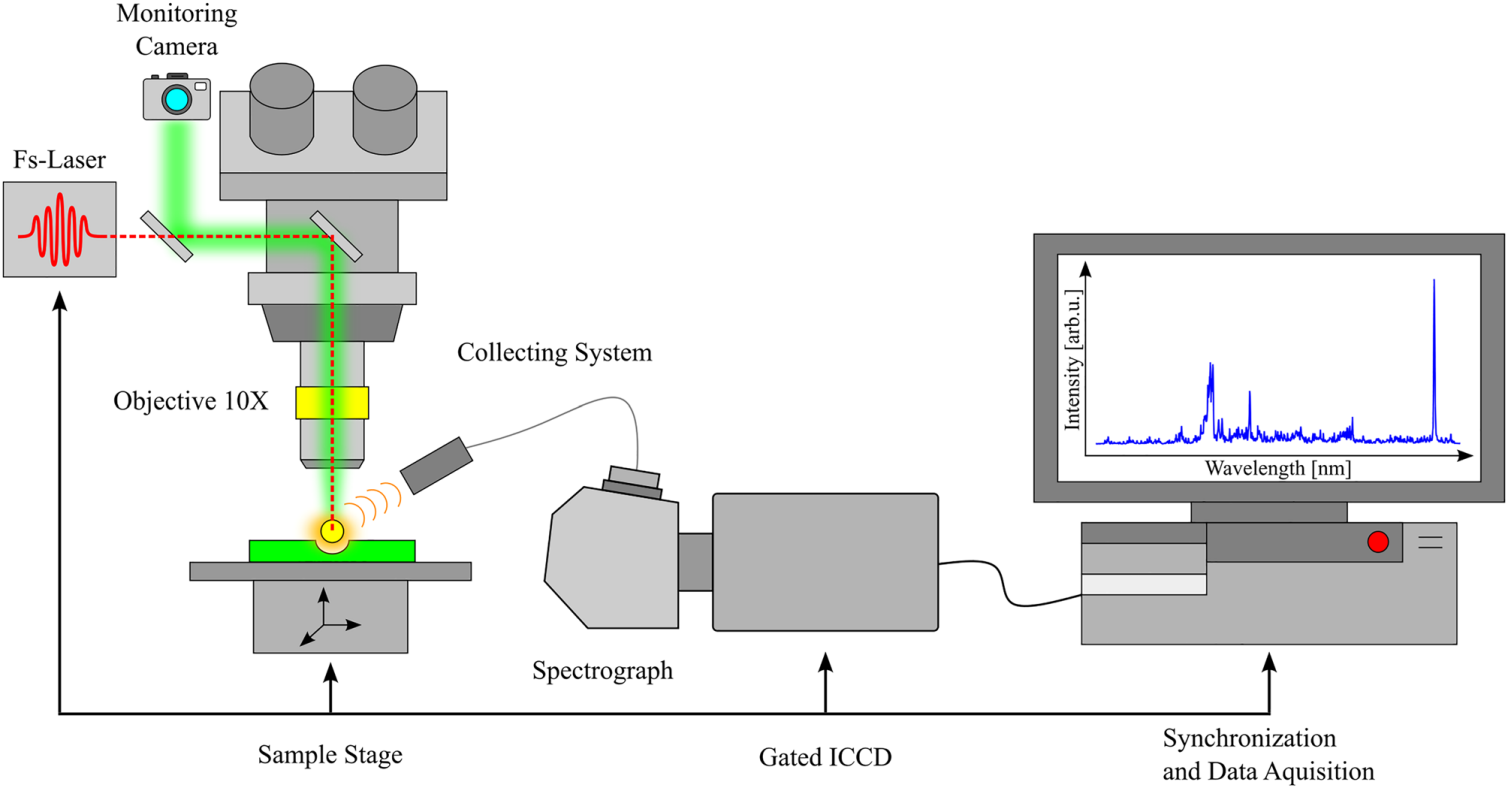
Schematic representation of the experimental setup.

Laser pulses with a temporal width of 30 fs at a central wavelength of 785 nm and a repetition rate of 1 kHz were generated by an amplified Ti:Sapphire laser system (Femtolasers, Femtopower Pro). The repetition rate is reduced down to single shot by using the Pockels cell (as an electro-optic pulse selector) inside the amplifier. The linearly polarized light was guided to a high precision home-build pulse-shaper^30,31^, where the overall dispersion of the optical elements up to the sample surface was compensated using a two-photon photodiode signal as feedback. Between the laser system and the microscope platform, we have several optical components to vary and record the energy of the pulses. Figure 1 shows a schematic diagram of the fs-LIBS setup used in this study. The main components on the microscope platform are a monitoring camera for sample positioning, a stepper-motor controlled XYZ translation stage (PI miCos), and a tilt stage to make the surface of the sample parallel to the x and y movement directions of the stage. A 10X Mitutoyo Plan Apo objective with a numerical aperture (NA) of 0.28 and 34 mm working distance focuses the laser pulses to a 3.5 µm measured beam radius (at $1/{e}^{2}$ of intensity) on the sample. With a Rayleigh range below 12 µm, the plasma is created on the thin biological sample, producing minimal ablation from the material of the substrate. The measurements were performed at room temperature in air atmosphere, using an energy per pulse of $7\pm 0.5 \mu \mathrm{J}$ and a corresponding peak intensity of about $5\times {10}^{14}\mathrm{ W}/{\mathrm{cm}}^{2}$, which assures a good signal-to-noise ratio with very low spectral contribution from the substrate.

For each selected location on the sample with pre-identified tissue type, we ablated a 10 × 10 matrix in a single-shot regime of the laser; after the spectrum of the plasma created by each laser pulse was recorded, the sample was moved by a spot-to-spot distance of 25 µm.

The emitted light from the laser-induced plasma was collected at a NA of 0.22 by a system of two fused silica lenses positioned at 45° close to the microscope objective and transported through an optical fiber to the 50 µm entrance slit of the spectrometer (L.O.T. Oriel Multispec MS125). For this experiment, we used a grating with 400 lines/mm and 500 nm blaze (L.O.T. Oriel 77417), which assured a spectral resolution of about 1 nm. An intensified Roper Scientific PIMAX ICCD was attached to the spectrometer to record the spectra. Wavelength calibration was performed with a calibration lamp (L.O.T. Oriel Pen Ray 6035 Hg(Ar)) without intensity calibration.

The data acquisition was synchronized with the laser system. The camera’s programmable timing generator controls the delay and the exposure time (gate) of the recording with ns precision. For the fs-LIBS emission, we used a 23 ns delay after the laser pulse and a gate time of 500 ns to suppress the supercontinuum emission of the laser pulse and the broadband background from continuum bremsstrahlung. Each spectrum was saved and labeled accordingly to the tissue type.

### Sample preparation and measurement procedure

**Figure 2:**
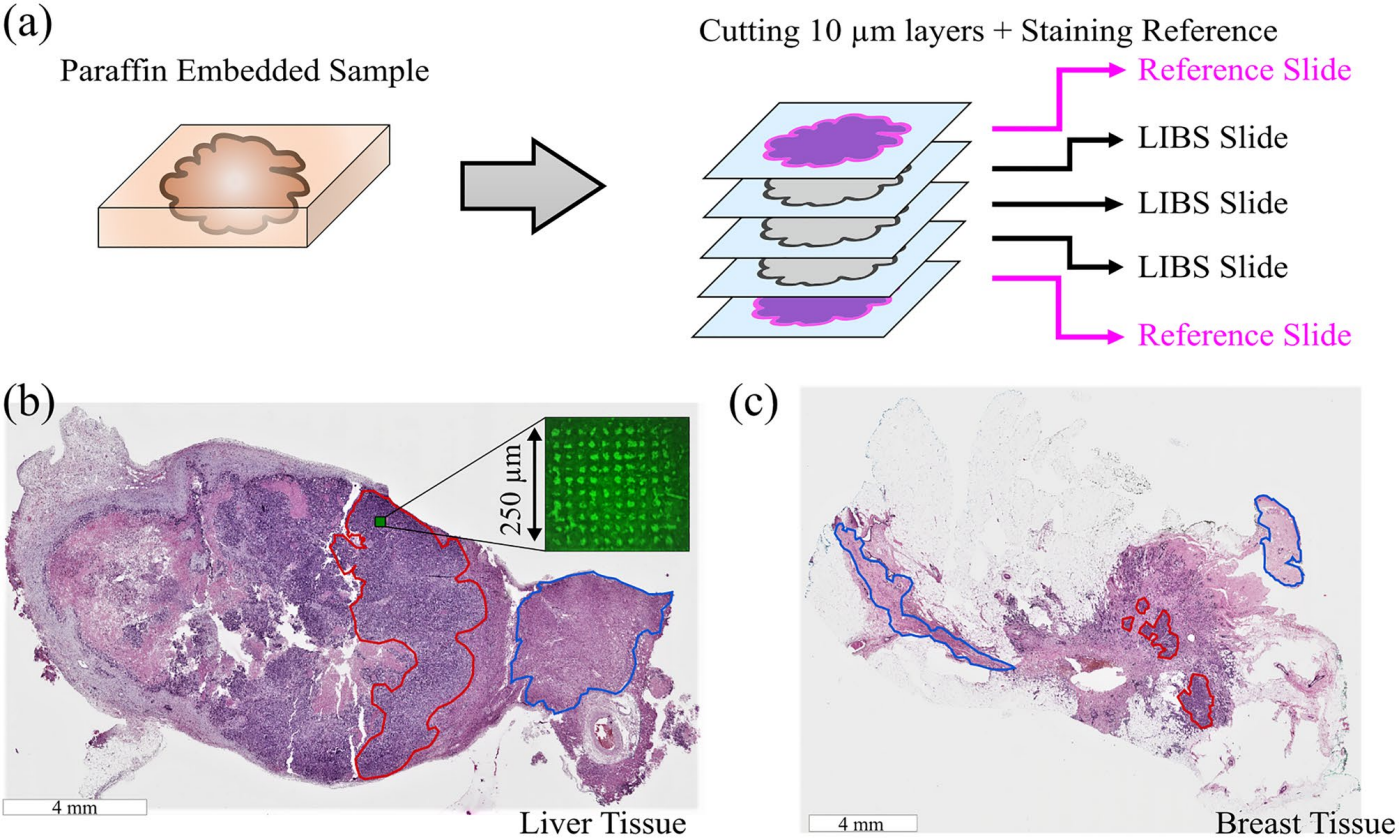
Schematic representation of sample preparation in (**a**) and microscopy images of the two tissue types (liver in (**b**) and breast in (**c**)). The red markings in (**b**) and (**c**) indicate pure Tumor regions, while the blue markings show pure, Healthy regions. The remaining areas contain a mix of Tumor and Healthy cells. The inset in (**b**) shows the image of an ablated matrix superimposed on the corresponding area in the reference image.

**Figure 3:**
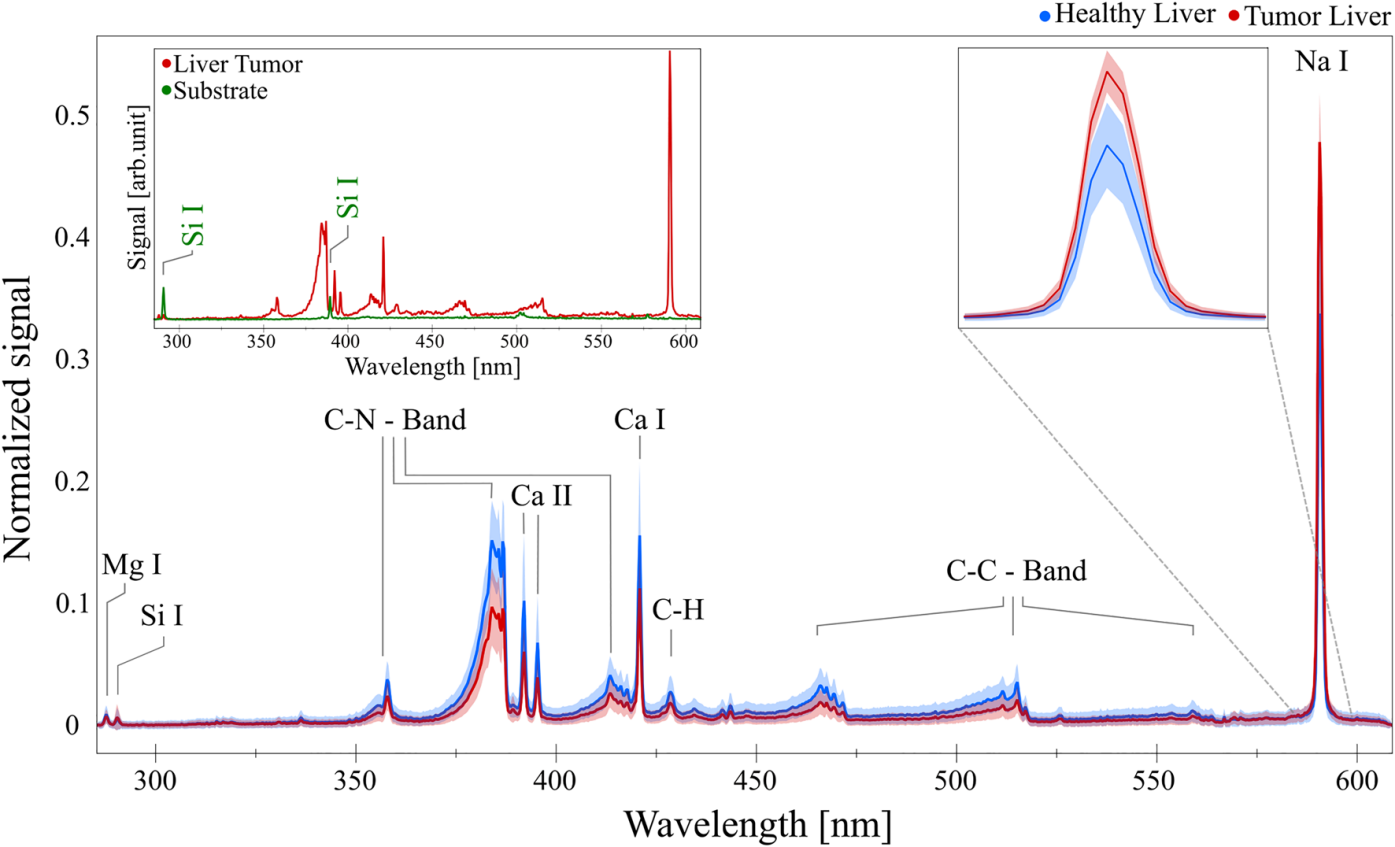
Preprocessed (baseline subtraction and vector normalization) liver spectra of both classes (red = “Tumor”, blue = “Healthy”, including the respective signal distribution of each wavelength within the dataset indicated by the correspondingly colored shades. The insertion on the top-left shows an average of 100 spectra recorded from the empty substrate (quartz, green) and tumor liver tissue (red) and the one on the top-right shows an enlarged part of the spectrum for better visualization of the Na line.

Formalin-fixed and paraffin-embedded tissue samples of human liver containing a metastasis of colorectal cancer, breast tissue with primary tumor, as well as a lymph node with metastatic breast cancer were investigated. Serial 10 µm thin sections were prepared from each paraffin block by a microtome and the paraffine was removed by subsequently dissolving it with xylene, alcohol, and water according to standard protocols^32^. The outermost slices of the stack were stained with a standard H&E (Hematoxylin and Eosin) protocol to identify regions of healthy and cancerous tissue (Fig. 2a). This way, each set of two or three LIBS slides was encapsulated by a pair of reference slides and we can use classical pathological analysis by optical inspection on the stained outer slices to select the corresponding locations on the inner slices for spectral investigation with high certainty. The slices with lateral sizes of around 2 × 1.5 cm were placed on a microscopy slide. In LIBS analysis on very thin samples, an important issue is the choice of substrate^28^, which can present strong spectral lines of impurities^22^. For this reason, we have chosen high purity quartz glass (Plano GmbH) substrates, where the only emission lines present in the measured spectral range are those of silicon (Si), as it can be seen in the insertion in Fig. 3. As silicon is present in negligible amounts in the breast and liver biological tissues analyzed^33,34^, we removed spectra with significant Si signals because these can only originate from the substrate. Another way of avoiding substrate problems (and getting higher signals) would be thicker slices, but they are difficult to obtain and manipulate by this procedure.

Table 1 presents for each patient the number of LIBS samples and the total number of recorded spectra in the tumor and healthy areas. The samples from patient 3 are only breast tumor tissue grown in lymph nodes (no healthy breast tissue).

**Table 1:** Number of LIBS samples and the total number of acquired spectra (before preprocessing) for each patient.

Tissue type	Origin	No. LIBS samples	No. spectra healthy	No. spectra tumor
Breast with primary cancer	Patient 1	2	2000	1750
	Patient 2	2	6400	4600
Lymph node with breast cancer metastasis	Patient 3	3	–	5000
Liver with metastasis	Patient 4	4	8100	8100

Figure 2 shows images of two reference slides from both types of tissues (liver and breast). Tumorous regions are represented by a dark violet color (Fig. 2b and c, red marked area), while healthy regions are indicated by lighter color (Fig. 2b and c, blue marked area). Spectral measurements were limited to the marked areas to ensure the correct identification and labeling of the tissue type.

The resulting spectra are labeled with the corresponding class (“Tumor” or “Healthy”) for later analysis. To avoid bias (during ML) based on experimental conditions, a comparable number of spectra from each type of tissue was recorded during each experimental session. In our approach, the ML is a binary classification and its goal is to sort given spectra into the classes of “Tumor” and “Healthy”. It must be mentioned that any differentiation of tumor stages is not considered during the evaluation. The class “Healthy” also does not regard the tissue type or cell types but just the absence of tumor cells. The processing of the spectra and the analysis through ML algorithms were done through the software application for data mining, Orange version 3.32.0^35^.

### Ethics declaration

Formalin-fixed and paraffin-embedded tissue samples of human liver containing a metastasis of colorectal cancer, breast tissue with primary tumor, as well as a lymph node with metastatic breast cancer were retrieved from the archive of the Institute of Pathology Nordhessen (Germany) in compliance with a vote of the ethical committee for scientific research (provided by Hessische Landesärztekammer, FF61/2014). In accordance with this approved vote, we have been using non-vital tissue samples aged more than 10 years comprising excess material that is not of diagnostic relevance anymore. In such cases, an additional patient’s informed consent is not needed.

## Results and discussion

### Proof of principle

As a first step, the training and testing of the ML algorithm is limited to the spectra from liver samples. They include large areas of tumor and healthy tissue, giving high certainty for correctly identify and label the recorded spectra. This gives a baseline for the performance of our proposed method before extending the analysis to more complex cases.

A sequence of preprocessing steps prepared the acquired data for the subsequent analysis. At first, unsuitable spectra were sorted out using the “the local outlier factor” widget inside the Orange software which measures the local deviation of a given recorded spectrum with respect to its neighbors (we used a contamination factor of 5% for 25 neighbors). In addition, spectra containing a strong Si I spectral line at 288.34 nm indicate that the laser pulses ablated also the fused silica substrate and all the spectra containing at this wavelength a signal higher than the background plus the maximum random noise fluctuations for this spectral range were also removed. For the remaining data, three adjacent spectra were averaged, effectively reducing the dataset by a third. Baseline subtraction and vector normalization of the spectra^36^ were applied to correct minor deviations from the recording process, like fluctuations in the energy of the laser pulses. The full range of the ICCD’s native 1024 pixels was not used, but 30 of them at each end were removed due to some vignetting at the corners of the CCD sensor. Finally, the liver dataset contained a total of 2392 averaged spectra labeled as “Tumor” and 2544 as “Healthy” with 964 features (pixels that correspond to the wavelengths in the recorded spectra).

Laser-induced emission spectra recorded for both classes are presented in Fig. 3. They display atomic lines^37^ as well as molecular bands^38^. The lines and bands identified agree with the emission spectra of biological tissue presented in other publications^15,16,38,39^. It is worth mentioning that in the case of fs-LIBS, the molecular emission lines are stronger than in the case of ns pulses, as other authors also pointed out^27,28^, and that the nature of these bands is the fragmentation of organic molecules and not the reaction of carbon with nitrogen in the ambient laboratory atmosphere, as discussed in Ref.^40^. The Ca II double line at 396.8 nm and 393.4 nm is the only ionic contribution in the spectrum. A visible difference in the spectra (illustrated in Fig. 3) between the two categories is the intensity ratio of the Na line, but discrimination only on this line is not as accurate as using ML algorithms considering the full spectral information, which can identify it in more detail and provide better classification.

The following evaluation of different ML algorithms was performed with the standard of tenfold cross-validation on the liver dataset. The performance of the algorithms is judged by their ability to detect each class separately and the overall percentage of correct decisions, namely the Classification Accuracy (CA).

**Figure 4:**
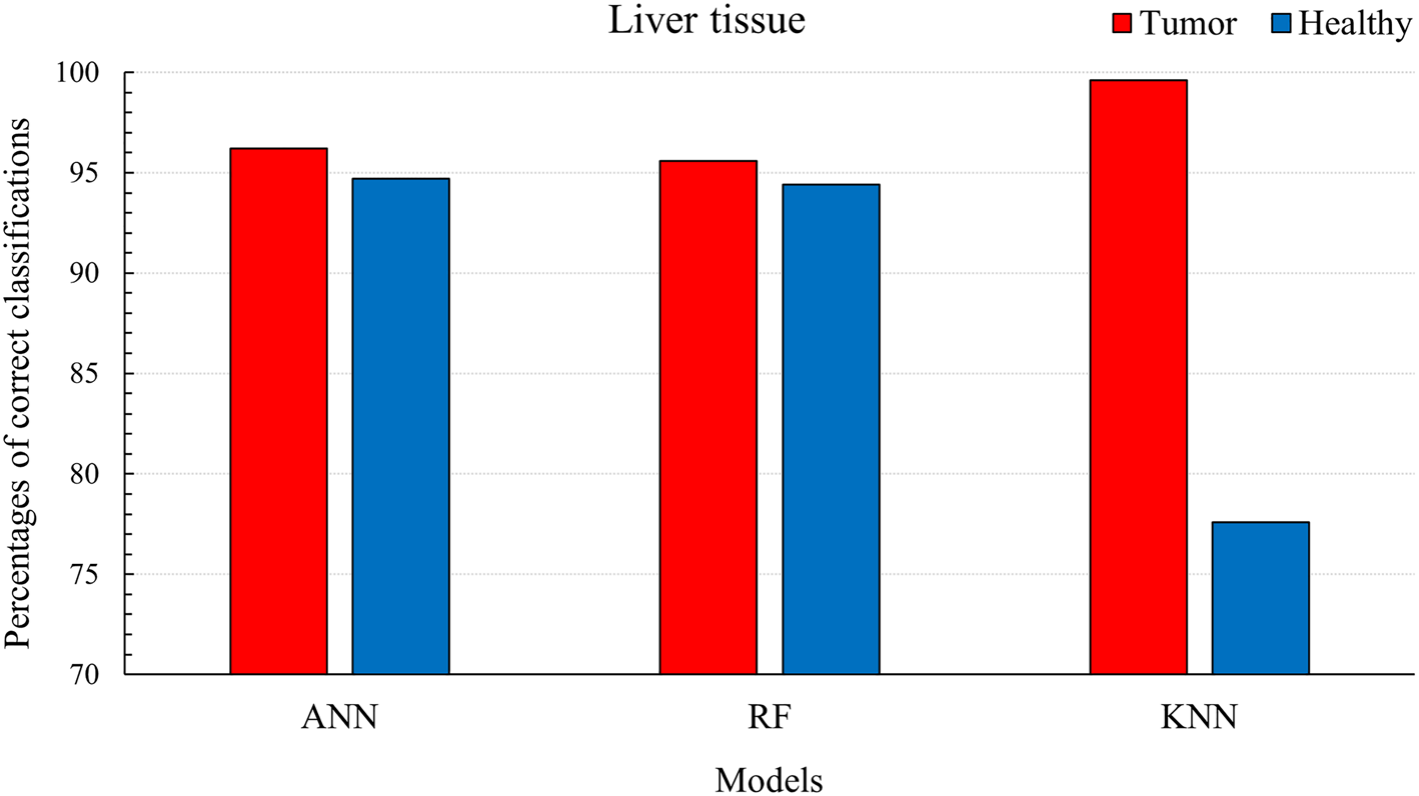
Percentages of correct classifications using Artificial Neural Networks, Random Forest and K-Nearest Neighbor for Tumor and Healthy liver tissue.

Several algorithms with different complexity and structures were chosen to analyze the data: naïve Bayes, Decision Tree, Support Vector Machine, K-Nearest Neighbors, Random Forest and Neural Networks. Their CA score is above 0.85 and indicates a very good ability of all models to discriminate the tissue classes accurately. The top three algorithms, namely Artificial Neural Networks (ANN), Random Forest (RF) and K-Nearest Neighbor (KNN), were optimally adapted to the dataset by adjusting their internal parameters to obtain the highest percentage of correct classification. For ANN we used 512 neurons in one hidden layer, 250 trees in RF and 25 neighbors for KNN. The results of the trained algorithms are summarized in Fig. 4, where the bar plots represent the percentage of the correct classification. The ANN and RF models can detect both classes on the same level (having a CA score ~ 0.95), while the KNN is more sensitive toward tumors (CA score 0.88).

### Feature importance

The presented spectra show distinct characteristics, raising the question of which features (wavelengths) are relevant for the algorithm’s decisions. This section will show the analysis of spectral line importance for the discrimination.

**Figure 5:**
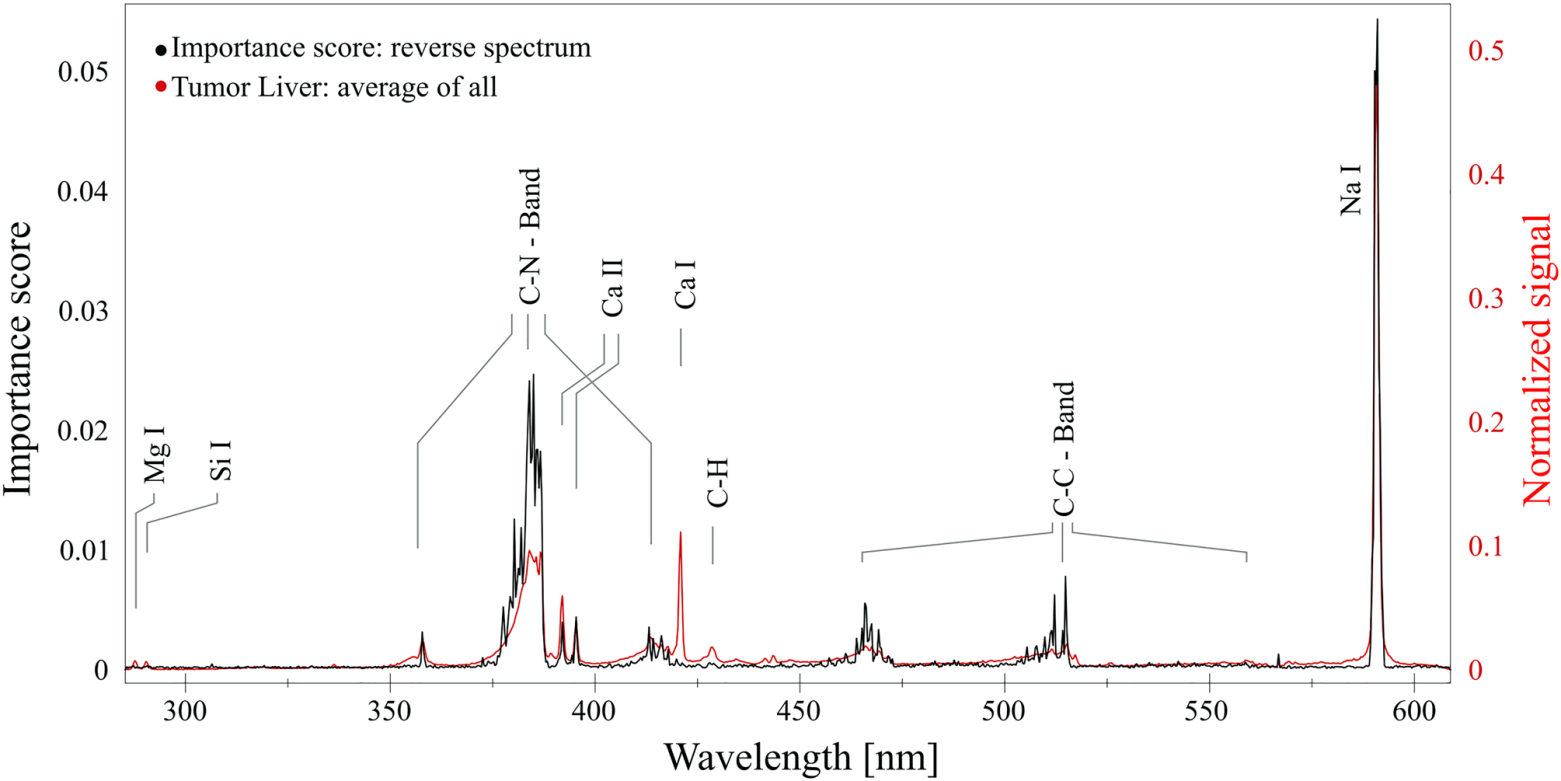
The reverse spectrum (importance score for each wavelength) of the Random Forest algorithm overlapped with the averaged recorded emission spectra for liver tumor.

A straightforward approach for this task is to reverse one algorithm in such a way that it uses the class (“Tumor” or “Healthy”) as input and calculates a “reverse spectrum”, where the intensity of each line describes the importance of the algorithm’s decision. The RF and its decision tree-based structure allows calculation of such a “reverse spectrum” via the mean decrease of impurity for all trees (effectively, how good is the dataset mapped onto each class for each tree and each wavelength)^41^. Figure 5 displays the results of the calculation. It is visible that the “reverse spectrum” resembles the ones recorded, although the intensities of the spectral lines differ significantly. The sodium line has the highest importance for the algorithm’s decision process, which is expected, as its divergent intensities were observed in the recorded spectra. The increase of the Na line emission in tumoral tissue has also been discussed in other publications^16,17^ and was attributed to the intracellular increase of Na resulting from a change in the Na^+^/H^+^ kinetics in an acidic extracellular environment. The C–N band has the second highest score and the second highest intensities in the real spectra and, together with the C–C bands, displays the importance of molecular signals for decision making. However, it is essential to mention that these observations only apply to the RF model and are not necessarily applicable to the other models. Nonetheless, it provides insight into the decision-making process and displays that the algorithms use spectral lines to identify the classes.

We emphasize that the use of ultrashort pulses in LIBS also allows the use of molecular emission bands in the analysis, unlike ns-LIBS, where the differentiation is mainly based on atomic and ionic lines. Reducing the dimensionality of the spectra^42^ by using as input in ML different combinations of atomic lines or molecular bands generate a lower CA than using the entire spectral information (results not in the scope of this work), so our test will focus on better classifications.

### Reproducibility

**Figure 6:**
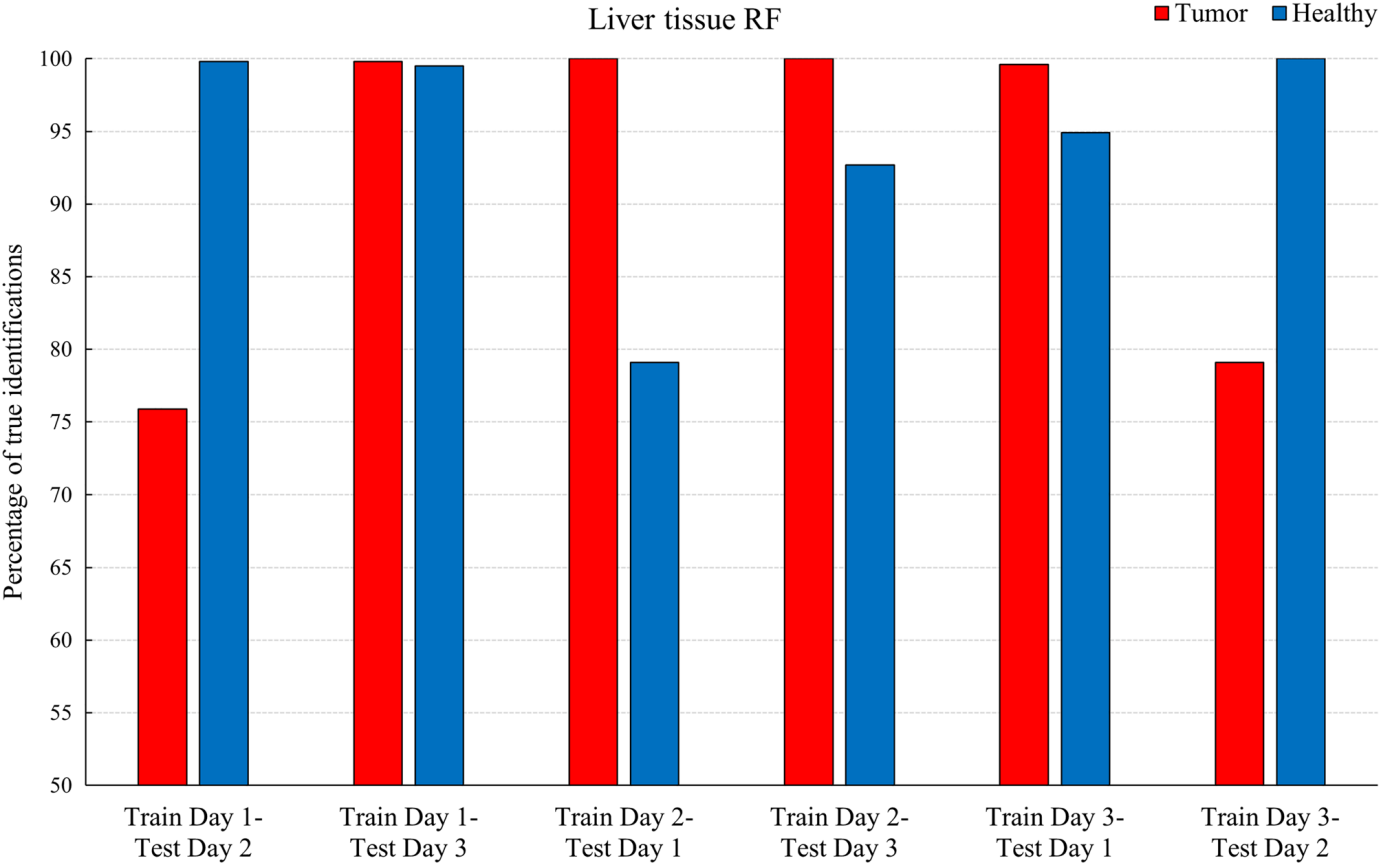
Reproducibility of the measurements on liver tissue. Percentage of correct classifications when the Random Forest algorithm was trained in one day and tested on the data measured on a different day.

After observing the ML algorithms’ ability to identify tumor tissue accurately and proving that the decision making is based on spectral lines with varying degrees of importance, the next step is to investigate the reproducibility of fs-LIBS and to check its application as an analytic tool. The ablation processes are complex and sensitive to various external parameters. This complexity is further increased for LIBS investigations on biological tissue, as its elemental composition is highly heterogeneous. To avoid this problem, in many studies, broad focal spots are used for irradiation, thus reducing the spatial resolution and increasing the collateral damage^13,43–45^, or they use a large number of spectra for averaging^14,15,18^. Fs-LIBS has the advantage of recording more reproducible spectra compared to traditional ns-LIBS due to its deterministic nature of ionization and the absence of the laser-plasma interaction during the laser pulse. The aspect of reproducibility and consistency of the dataset is approached in the following: on the same sample of liver tissue, we performed measurements on three different days where we optimized the set up for each day individually. Therefore, the spectra should ideally be the same, assuming that every ablated material within one tissue type contains the same elemental composition. Consequently, training ML algorithms on the spectra recorded in one day while testing them on the ones from another day should yield the same results. Thus, the three optimized models, KNN, RF and ANN, were tested for all possible combinations of the three daily spectra subsets. All these algorithms show good day-to-day reproducibility, with percentages of correct classification above 75% and in the following we will discuss the results obtained using RF. Figure 6 displays the correct predictions for the Tumor and Healthy classes using the RF algorithm.

The different bars display a visible deviation of the algorithm’s performance. For example, the RF ability to detect tumor tissue varies in the case of „Train Day 1-Test Day 2” versus “Train Day 1-Test Day 3” by about 20%. However, deviations are to be expected since many factors, like slightly different adjustments of the setup and the inhomogeneity of biological tissue, will impact the performance of the models. Furthermore, the model can accurately identify each class with percentages higher than 75% and consistent CA scores (not shown here) above 0.88, indicating consistency within the dataset.

### Algorithms’ performance on unknown data

**Figure 7:**
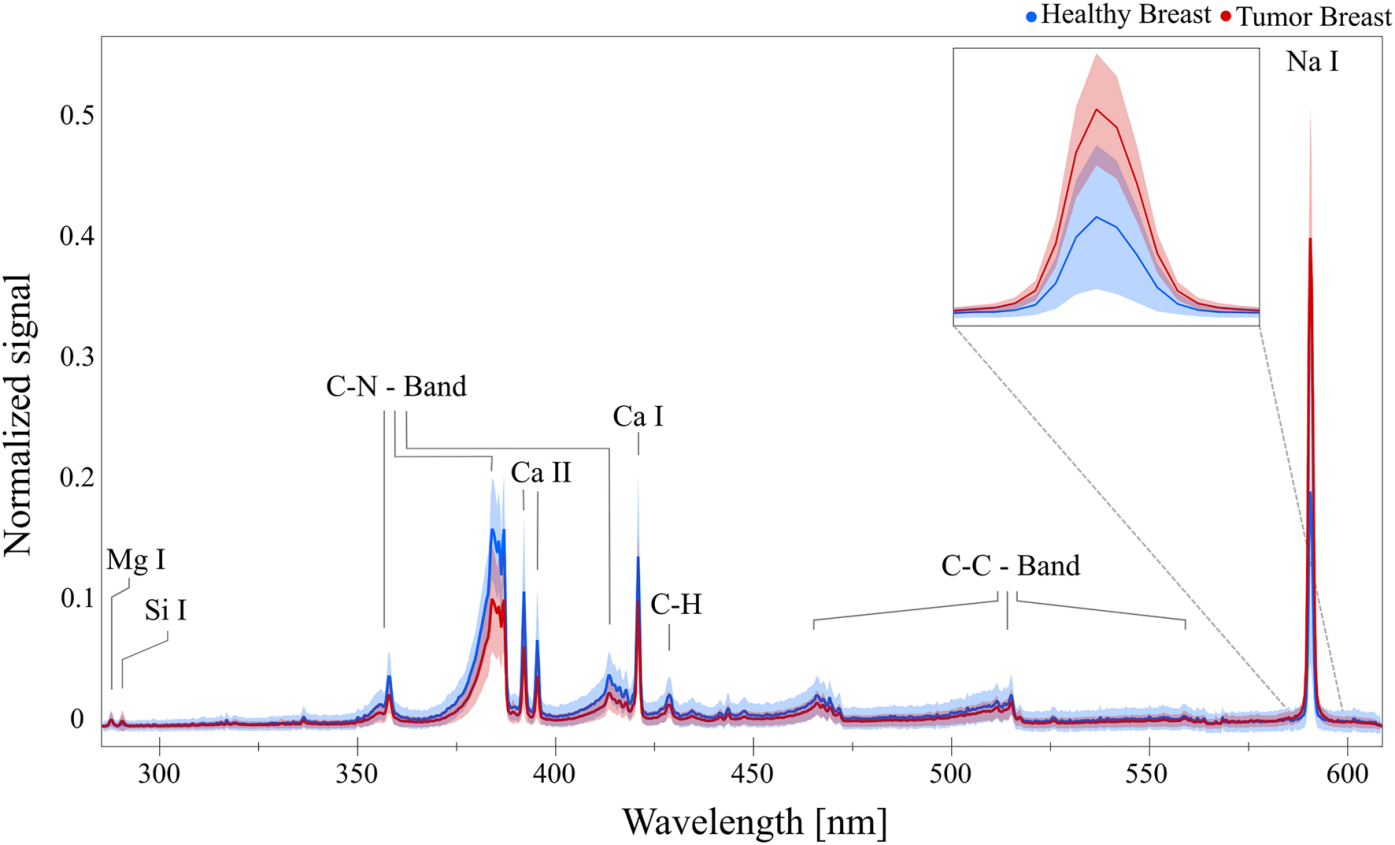
Preprocessed (baseline subtraction and vector normalization) breast spectra of both classes (red = Tumor, blue = Healthy), including the respective signal distribution of each wavelength within the dataset (colored shadows). The insertion on the top-right shows an enlarged part of the spectrum for better visualization of the Na line.

Breast tissue is one of the most heterogeneous ones^46^. On the pathological samples we analyzed, the areas with clear identification of the tumor and healthy tissue were smaller than in the case of the liver tissue (as seen in Fig. 2). This made the measurement procedure more difficult and increased the chance of mislabeling. The breast samples were provided from three different patients and thus allowed us to investigate the aspect of generalization compared to the liver dataset, which was only from one patient. The breast tumor and healthy tissue spectra are similar to the liver and are displayed in Fig. 7.

The assignment of spectral lines and bands, as well as preprocessing, model selection, and fine tuning, were performed analogously to the liver data set. The three optimized models (KNN, RF and ANN) perform on a very high level with consistent CA scores above 0.85 (KNN = 0.85, RF = 0.92 and ANN = 0.91) when the whole set of spectra for the three patients is analyzed.

**Figure 8:**
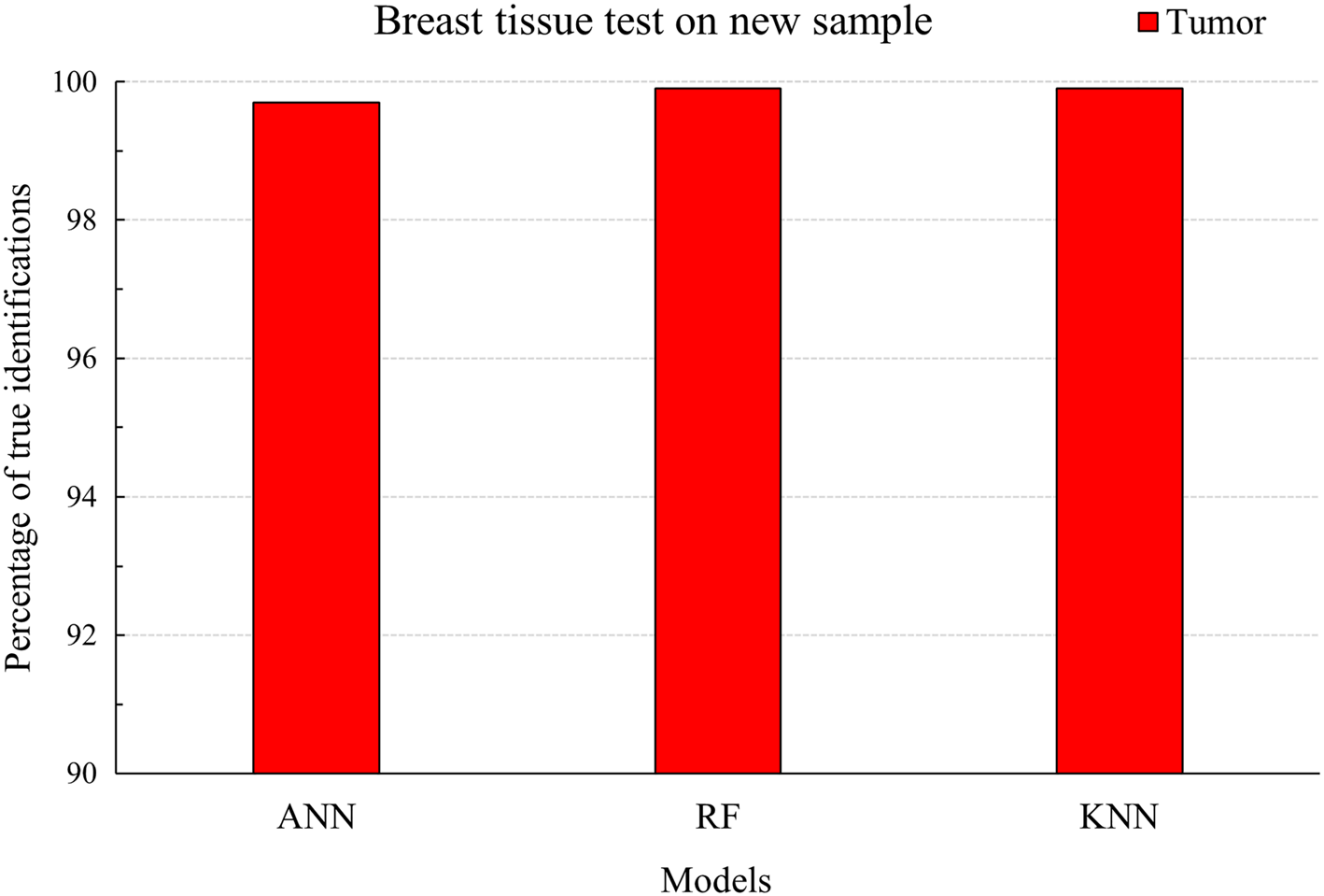
Percentages of correct classifications of metastasis when the algorithms were trained on primary tumors from other patients.

In the sense of generalization, we tested the ability of the algorithms to correctly classify new data coming from unknown patients. Within the scale of this project, we used the dataset from the two patients with primary tumors for the training process and tested the algorithms’ ability to identify the metastasis tumor on the third patient. The training pool consisted of spectra from both classes, while the test set contained only tumor tissue spectra. We used 1985 Tumor and 2558 Healthy spectra for the training phase, while the test on the new patient was performed on 1590 Tumor spectra. Figure 8 displays the results of the correct classifications from the three models.

The percentage of 99.9 (KNN and RF) and 99.7 (ANN) display a surprisingly excellent prediction rate and suggest the potential for generalization. However, since the training pool consisted of two patients and the test pool of one patient with breast cancer cells in lymph nodes, a larger pool of patients is required for more definite conclusions.

## Conclusions

It has been demonstrated that fs-LIBS can be used with very thin samples on a quartz glass substrate and that the signal produced is strong enough to differentiate tumorous from healthy liver tissue with a high spatial resolution. We correctly identified the tissue type using a mix of adjacent microtome-cut LIBS- and reference- slices. Random Forest and the Artificial Neural Networks algorithms performed the best, correctly classifying both tumor and healthy liver tissue in more than 94% of cases. In this case, the K-Nearest Neighbor algorithm produced excellent identification of tumor tissue but had modest results in the case of healthy tissue. By using the RF algorithm, the feature importance was calculated, leading to the conclusion that molecular bands and sodium line are prioritized in the decision process. The presence of the molecular lines represents one of the advantages of using ultrashort lasers, which produce a good spectral signature after the ablation with low-energy pulses. This avoids massive dissociation of the molecular biological material as in the case of ns-laser pulses, where the tissue type discrimination is based only on the atomic or ionic lines^20,22,43^. The possibility of using this technique in a new, unknown patient was explored on breast samples with very good classification accuracy.

In the future, in addition to increasing the number of patients and studying the influence of the sample preparation, we want to investigate the pulse length dependence for tissue differentiation. In order to further increase the identification accuracy, alongside the improvement of the signal-to-noise ratio by using double time-delayed femtosecond pulses^25^ more complex numerical algorithms^47^ will be used. Possible applications in the intraoperative field should consider the availability of laser sources, where high repetition rate fiber lasers with longer pulse durations seem to be promising^48^ and can also be easily integrated in endoscopic devices.

We expect that this method can be used in situ/in vivo as a complementary method to the standard pathological analysis.

Considering the spatial accuracy of ablation with femtosecond laser pulses and the low sample volume required, we believe that intra-surgical information about the nature and extent of the tumor could be obtained after the identification algorithms were trained on biopsy samples from the same patient. In the distant future, when fs-lasers may be used as surgical tools, this method could help distinguish healthy from diseased tissue directly during incision.

## Data Availability

The datasets during and/or analyzed during the current study available from the corresponding author on reasonable request.
